# Improved liver disease prediction from clinical data through an evaluation of ensemble learning approaches

**DOI:** 10.1186/s12911-024-02550-y

**Published:** 2024-06-07

**Authors:** Shahid Mohammad Ganie, Pijush Kanti Dutta Pramanik, Zhongming Zhao

**Affiliations:** 1 AI Research Centre, Department of Analytics, School of Business, Woxsen University, Hyderabad, Telangana 502345 India; 2 School of Computer Applications and Technology, Galgotias University, Greater Noida, Uttar Pradesh 203201 India; 3 Center for Precision Health, School of Biomedical Informatics, The University of Texas Health Science Center at Houston, Houston, TX 77030 USA

**Keywords:** Liver disease, Disease prediction, Ensemble learning, Boosting, Bagging, Voting, Gradient boosting

## Abstract

**Purpose:**

Liver disease causes two million deaths annually, accounting for 4% of all deaths globally. Prediction or early detection of the disease via machine learning algorithms on large clinical data have become promising and potentially powerful, but such methods often have some limitations due to the complexity of the data. In this regard, ensemble learning has shown promising results. There is an urgent need to evaluate different algorithms and then suggest a robust ensemble algorithm in liver disease prediction.

**Method:**

Three ensemble approaches with nine algorithms are evaluated on a large dataset of liver patients comprising 30,691 samples with 11 features. Various preprocessing procedures are utilized to feed the proposed model with better quality data, in addition to the appropriate tuning of hyperparameters and selection of features.

**Results:**

The models’ performances with each algorithm are extensively evaluated with several positive and negative performance metrics along with runtime. Gradient boosting is found to have the overall best performance with 98.80% accuracy and 98.50% precision, recall and F1-score for each.

**Conclusions:**

The proposed model with gradient boosting bettered in most metrics compared with several recent similar works, suggesting its efficacy in predicting liver disease. It can be further applied to predict other diseases with the commonality of predicate indicators.

## Introduction

Liver disease is a significant global health burden, accounting for two million deaths annually, with approximately two-thirds in men [1]. Liver-related fatalities constituted 4% of the deaths observed in the current century [2]. Liver disease encompasses a spectrum of conditions, including fatty liver disease, cirrhosis, and hepatocellular carcinoma, which can lead to liver failure and death. The primary factors contributing to the development of liver disease are the frequent and prolonged consumption of drugs and alcohol, as well as the presence of obesity and diabetes [3]. Intervention and early diagnosis are essential for enhancing patient outcomes in liver disease. However, the sensitivity and specificity of conventional diagnostic techniques, including liver function tests and biopsies, are currently limited.

Machine learning (ML) has emerged as a promising tool for improving the diagnosis and prognosis of human diseases, including liver disease. ML algorithms can empower the analysis of large but complex clinical data, often including patient demographics, family history, patient medical records, laboratory results, and imaging findings, to identify patterns and relationships associated with liver disease. This information can then be used to develop predictive models for early disease detection and risk stratification. Several studies have investigated the application of ML for liver disease prediction using clinical data [4, 5]. These studies have explored various ML algorithms, including support vector machines (SVMs), random forests (RFs), and artificial neuron networks (ANNs). However, the performance of these algorithms can be affected by factors such as data quality, feature selection, and model parameters.

**Fig. 1:**
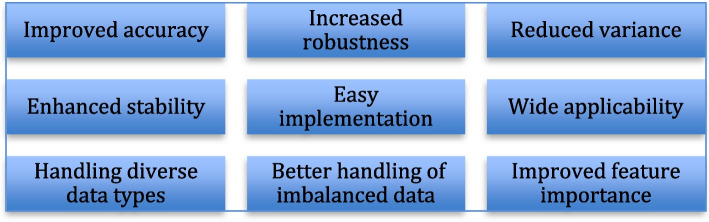
Advantages of ensemble learning approaches

One of the most powerful ML approaches for medical diagnosis is ensemble learning. Ensemble methods combine multiple base learners to create a single, more robust model. This can improve the accuracy and generalizability of predictions compared to individual models. Ensemble learning has numerous advantages compared to conventional ML methodologies, rendering it a potent methodology for enhancing prediction efficacy across diverse workloads. Some of the notable advantages of ensemble learning are summarised in Fig. 1. Ensemble learning methods are becoming increasingly popular for more precise disease prediction [6–10]. Considering its success in other disease prediction, ensemble learning has also been explored to predict liver disease because this is a major disease type with a large amount of data [11].

Several ensemble learning strategies have been developed. Among them, the most common ones include bagging (e.g., BDT (bagged decision tree), RF (random forest), ET (extra trees), etc.) [12], boosting (e.g., AdaBoost, GB (gradient boosting), and XGB (eXtreme gradient boosting)) [13], and stacking/voting (e.g., LR, DT, SVM, etc.) [14]. The selection of an ensemble technique should be determined by the particular problem at hand, the characteristics of the dataset being used, and the computational resources that are accessible.

This paper aims to extensively evaluate the ensemble learning methods for liver disease prediction and find the best-performing one. The main works in this paper are highlighted as follows:

- An EDA is conducted to augment the dataset under consideration so that it can be utilized more effectively in experiments.
- Different subsidiary methods are employed, such as data sampling, standardization, normalization, hyperparameter tuning, and feature selection.
- Nine ensemble algorithms are applied for prediction model development.
- The model’s performances with the considered ensemble algorithms are exhaustively evaluated and compared using several performance metrics.
- The best performance of the proposed model is compared with other recent research works.

The remainder of the paper is structured as follows. Related work is discussed in Sect. 2. Section 3 briefly discusses the considered ensemble learning algorithms and the research methodology adopted in this paper. Details of the dataset and data preprocessing are discussed in Sect. 4. The details of the experimental setup are described in Sect. 5. The comparative analysis of the performance of the ensemble learning algorithms, along with other similar works, is presented in Sect. 6. The conclusion of our work, mentioning the limitations and future scopes, is given in Sect. 7. The acronyms used in this paper are listed in Table 1.

**Table 1:** List of abbreviations used in this paper

Abbreviation	Full name	Abbreviation	Full name
A1DE	Average one dependency estimator	IQR	Interquartile range
AdaBoost	Adaptive boosting	KDE	Kernel density estimation
ADASYN	Adaptive synthetic	kNN	k-nearest neighbors
ANFIS	Adaptive euro-fuzzy inference system	LASSO	Least absolute shrinkage and selection operator
ANN	Artificial neural network	LD	Liver disease
AUC	Area under the ROC curve	LGBM	Light gradient-boosting machine
BDT	Bagged decision tree	LR	Logistic regression
BUPA	British United Provident Association Ltd	LDPD	Liver disease patient dataset
CART	Classification and regression trees	MLP	Multilayer perceptron
CCA	Correlation coefficient analysis	NB	naïve Bayes
CDT	credal decision tree	NLD	No liver disease
CHAID	Chi-square automated interaction detection	RepTree	Reduced error pruning tree
CHIRP	Composite hypercube on iterated random projection	RF	Random forest
CNN	Convolutional neural network	ROC	Receiver operating characteristic
DT	Decision tree	RotF	Rotation forest
EDA	Exploratory data analysis	RT	Random tree
ENRR	Elastic net regularised regression	SMOTE	Synthetic minority oversampling technique
ET	Extra trees	SVM	Support vector machine
EV	Esophageal varices	UCI	University of California, Irvine
Forest-PA	Forest by penalizing attributes	VIF	Variance inflation factor
GB	Gradient boosting	WBC	White blood cell
ILPD	Indian liver patient dataset	WEKA	Waikato environment for knowledge analysis
INR	International normalized ratio	XGB	eXtreme gradient boosting

## Related work

The rise of ML has led it to be applied in various application areas, including diagnoses and predictions of diseases [15–17]. Pasha et al. [18] offered a prediction model for liver disease. They also compared their model’s prediction accuracy with other ML algorithms like RF, LR and SVM. Mutlu et al. [19] built a CNN-based model to identify liver disease. For the experiment, they used two datasets, BUPA (from BUPA Medical Research Ltd^1^.) and ILPD. Both datasets are available in the UCI ML repository^2^^,^^3^. The model attained 75.55% and 72% accuracy for the BUPA and ILPD datasets, respectively. The authors also compared this model’s performance with other ML techniques such as NB, SVM, KNN, and LR.

Kalaiselvi et al. [20] experimented with different ML algorithms like kNN, DT and ANFIS to determine which is more appropriate for liver disease prediction. They used the ILPD, which is available at Kaggle^4^. It was observed that ANFIS performed best in terms of all the performance metrics. Thirunavukkarasu et al. [21] attempted to predict liver disease using classification algorithms like LR, kNN and SVM. The experimental results on the ILPD showed that LR and kNN achieved equal accuracy and were better than SVM; however, LR performed better in sensitivity and specificity. Velu et al. [22] experimented with NB and C4.5 DT on ILPD to predict liver disease. The latter achieved a better accuracy of 98.40% with the test dataset.

In ensemble ML, complex and more efficient models are built by combining diverse ML techniques to gain their combined advantages. This collaborative approach has been proven to be successful in the prediction, detection, diagnosis, and prognosis of different diseases [23–27].

Amin et al. [28] proposed an integrated feature extraction approach to predict liver disease. They applied different dimensionality reduction methods like PCA, FA, and LDA on ILPD. Various ML classifiers like LR, RF, KNN, SVM, MLP and ensemble were evaluated on the extracted features using 10-fold cross-validation. RF achieved the best performance with 88.1% accuracy, 85.33% precision, 92.3% recall and 88.68% F1-score on the integrated feature space. Afrin et al. [29] used ensemble learning to predict liver disease using various classification algorithms like LR, DT, RF, AdaBoost, kNN, LDA, GB, and SVM. They used the ILPD and applied LASSO to identify the most important features correlated to liver disease. When using all features, LR performed best, with an accuracy of 77.14%. However, DT performed the best with LASSO features with 94.29% accuracy. DT also had the highest precision of 92%, sensitivity of 99% and F1-score 96% based on LASSO features.

Dritsas and Trigka [30] compared various ML models (NB, LR, SVM, J48, RT, and RepTree) and ensemble methods (bagging, RF, RotF, AdaBoostM1, voting, stacking, MLP, and kNN) for liver disease risk prediction. They applied SMOTE and 10-fold cross-validation. It was found that the voting performed the best with an accuracy of 80.1%, a precision of 80.4 and a recall of 80.1%. Nahar [31] compared different ensemble methods (AdaBoost, LogitBoost, RF, and bagging with J48 and Reptree) for liver disease prediction. They used the ILPD for the experiment and the WEKA toolkit to build and evaluate the model. The authors analyzed the performance of the ensemble methods over multiple iterations, showing how accuracy improves with more models. They evaluated the models using accuracy, RMSE, TPR, FPR and ROC curve, providing a comprehensive model performance analysis. The results indicate that LogitBoost has the best accuracy of 71.53%. Kuzhippallil et al. [32] compared various ML classification models and feature selection techniques to predict liver disease. They used a genetic algorithm and XGB to select features. They evaluated various models, including LR, kNN, DT, RF, GB, AdaBoost, XGB, LGBM, and the stacking model. After feature selection and outlier removal, LGBM and the stacking model achieved the highest accuracy of 86%. To find a better potential solution for liver disease prediction, Naseem et al. [33] presented an extensive comparison of ten classifiers, viz. A1DE, MLP, NB, kNN, SVM, CHIRP, CDT, Forest-PA, J48, and RF. They experimented with two different datasets taken from the UCI ML repository (BUPA^5^) and the GitHub repository (SanikaVT^6^). For the first dataset, RF exhibited overall better performance, while for the second, SVM was observed as best.

Quadir et al. [34] proposed an ensemble ML approach using enhanced preprocessing techniques to classify liver disease. They applied various preprocessing techniques like imputation, balancing, scaling, and selection to improve the model’s performance. The authors applied six ensemble algorithms (GB, XGB, bagging, RF, ET, and stacking) and evaluated them on the preprocessed data derived from ILPD. The extra trees classifier achieved the highest testing accuracy of 91.82% for liver disease classification. Dalal et al. [35] proposed a hybrid XGB model for predicting liver disease. When evaluated, the proposed model achieved a significantly higher accuracy of 93.65% compared to the individual DT models like CHAID and CART. It also had better performance metrics like AUC and Gini coefficient. Bulucu et al. [36] conducted a study to predict liver disease from clinical data using ensemble learning methods like RF, J48, AdaBoost, GB and LGBM. They performed SMOTE oversampling to balance the classes before classification. The LGBM algorithm performed best with 98.8% accuracy, 98.1% precision, 99.4% recall and 0.98% kappa statistic in 10-fold cross-validation.

Edeh et al. [37] experimented with an ensemble model comprising MLP, Bayesian network, and QUEST for Hepatitis C prediction. They used the HCV data set^7^, which allowed them to integrate the clinical data and blood biomarkers. An accuracy of 95.59% was achieved by the ensemble model, which was better than the individual performances of the considered algorithms. A predictive ML model of clinical outcomes presented by Meng et al. [38] aimed to assess the progression of Alpha-1 antitrypsin deficiency associated with liver disease (AATD-LD). They applied a supervised stacking ensemble learning technique combining RF, ENRR, GB, and ANN-MLP. They further mapped the importance of the feature for better interpretability of the predictive model. The authors extracted liver patient data from the UK Biobank for the experiment. Bayani et al. [39] used the factors that have the most influence on the prediction of EV grades among cirrhosis patients. To select the most potent predictors of EV grades, the authors used Catboost and XGB. In the experiment on a dataset of 490 patients with cirrhosis, 100% precision was attained with the Catboost model, while the XGB model had 91.02% accuracy. Child score, WBC, vitalism K, and INR were the most significant factors for predicting EV grades among cirrhosis patients. Gupta et al. [40] conducted a comparison of various ML approaches, such as GB, XGB, and LGB, to forecast liver disease. The dataset utilized for this purpose was the ILPD. 63% was the highest level of accuracy attained using RF and LGB. To predict liver disease using ILPD, Hameed et al. [41] also implemented many ML techniques, including boosting methods such as AdaBoost and GB. The findings indicate that the DT, AdaBoost, and RF achieved the highest accuracy during training, whereas the RF achieved the highest accuracy (80.36%) during testing. Zhao et al. [42] considered single classifiers (SVM and Gaussian process) and ensemble classifiers (XGB, bagging, and RF) for predicting liver disorders. The prediction performance was evaluated through accuracy, balanced accuracy, precision, recall, and F1-score. Experimenting with the BUPA dataset, the best performance was achieved through RF with an accuracy of 80.35%. However, bagging turned out to be a better performer in terms of recall.

All the above-mentioned studies used some basic machine learning models along with one or two ensemble models for liver disease prediction. Due to this, an exclusive performance assessment of the ensemble learning methods could not be availed. In this study, we built models using boosting, bagging and voting. Since the aggregation method is the fundamental policy of both stacking and voting, we kept only voting in this study. We performed a comprehensive comparison, considering the algorithms from different families of ensemble learning. In the actual experiment, we considered five algorithms from each category; however, here, we report the top three performers for each category.

Furthermore, most previous works reported only limited evaluation metrics that are generally common, e.g., accuracy, precision and recall. In this paper, we conducted thirteen statistical measurements to show the effectiveness of the proposed model from different aspects.

## Research methodology

A synopsis of the research procedures undertaken and the ensemble learning methods implemented in the experiment are described in this section.

### Research workflow

**Fig. 2:**
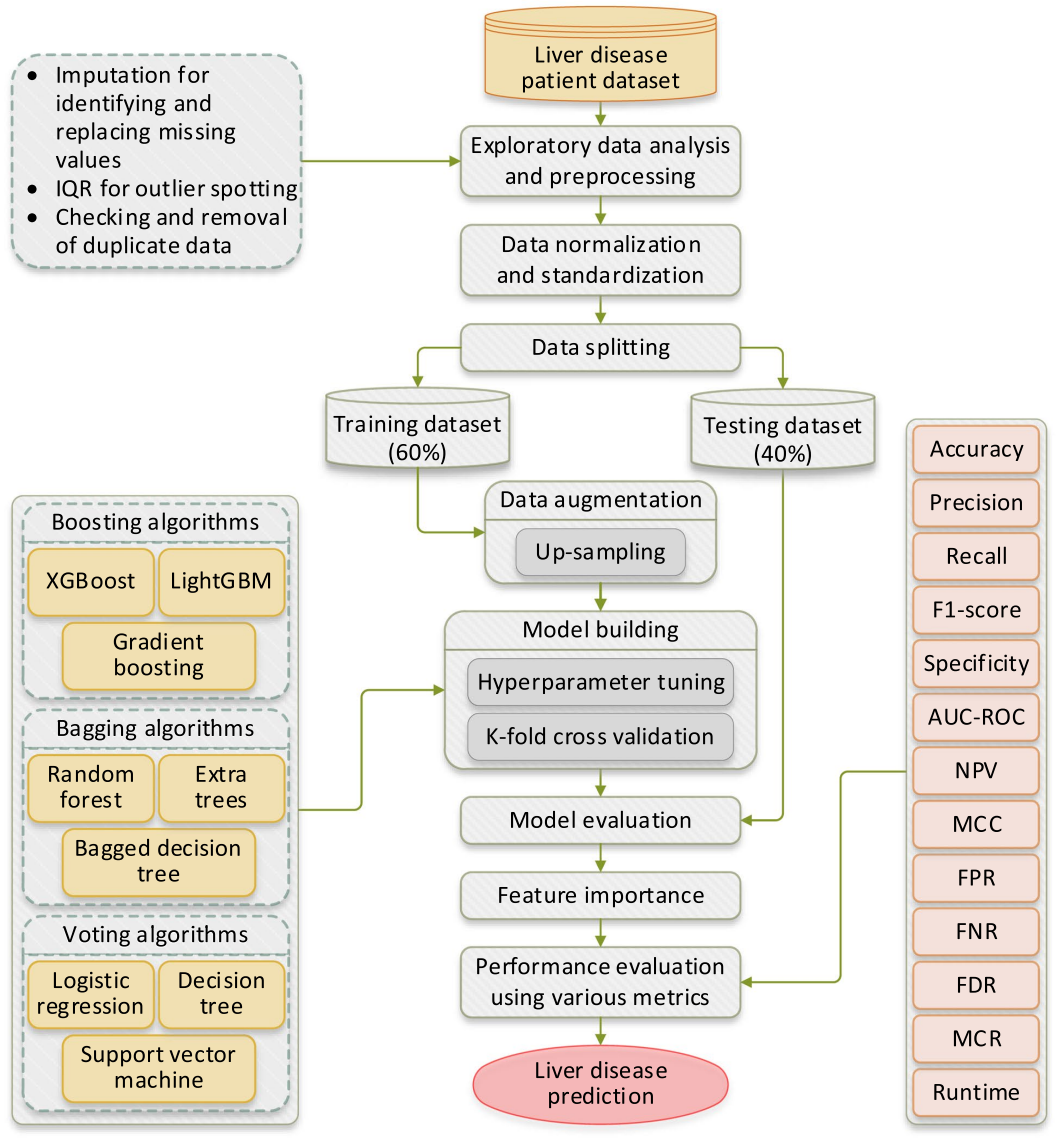
Proposed methodology for research work

Figure 2 summarises the workflow of this study. First, we performed EDA to assess and augment the quality of the considered dataset. Here, we searched for the missing values and replaced them by employing data imputation methods. Further, for spotting possible outliers, the IQR method was used. Besides, other libraries were used to check for corrupt and noisy data, if any, in the dataset. Afterwards, data sampling, normalization, standardization, hyperparameter tuning, and ranking of features as per their importance were made. To develop the prediction model, we used and compared nine ensemble algorithms. The results were assessed through various performance metrics. The ensemble algorithms were trained using 60% of the dataset, while the remaining 40% was allocated for testing and validating their effectiveness.

### Ensemble learning models

Ensemble learning is an ML methodology that improves the accuracy and robustness of predictions by combining multiple models, instead of relying solely on individual models [43]. The basic idea behind ensemble learning is that it can make up for the shortcomings of any single model by combining the strengths of different models, leading to better performance. A number of ensemble learning methods are suggested [44, 45]. We took into consideration the following ensemble learning techniques in this study:

#### Boosting

The boosting algorithm is a prominent method within the ensemble learning framework. Boosting methods involve an iterative training process where base models are trained, with increasing emphasis on misclassified examples in each iteration. In this manner, the emphasis is placed on rectifying errors committed by preceding models. Various boosting algorithms can be found in the literature [46, 47]. In this experiment, we considered the following three boosting algorithms.

- **XGBoost**: XGB is a popular boosting algorithm that combines different kinds of DTs (weak learners) to independently calculate similarity scores [48]. It is known for its speed, accuracy, and ability to handle complex data.
- **Gradient boost**: In this method, the weak learners undergo sequential training, while the weights of each estimator are adjusted individually before being added [49]. Predicting residual errors introduced by prior estimators, the GB algorithm attempts to minimize the discrepancy between predicted and actual values.
- **LightGBM**: LGBM is another popular boosting algorithm similar to XGB, but it is faster and more memory-efficient. It can manage sizable datasets while consuming less memory during model evaluation [50]. LGBM also has several features that make it well-suited for real-world applications, such as parallelization and out-of-core training.

#### Bagging

The bagging (bootstrap aggregating) technique entails the independent training of multiple base models on randomly selected subsets of the training data, with replacement. The final prediction is typically determined by taking the average (in the case of regression) or by voting (in the case of classification) the predictions generated by the base models. There are several bagging algorithms; however, in this study, the following methods gave the best results.

- **Bagged decision tree**: BDTs are the most basic implementation of the bagging technique [51]. They are generated by aggregating the predictions of numerous DTs trained on bootstrap samples of the data. Bagged DTs have demonstrated efficacy in mitigating variation and enhancing accuracy. However, it is worth noting that there is a potential for overfitting to the training data in certain cases.
- **Random forest**: RF is a more sophisticated bagging method that adds an element of randomness to the DT by randomly selecting a subset of features to examine at each split [52]. This further decorrelates the trees and can better the overall performance of the ensemble.
- **Extra trees**: ET is another kind of bagging that employs a different splitting rule for the DTs than standard bagging does [53]. Instead of employing a conventional approach of finding the optimal split at each node, additional trees adopt a randomization technique by randomly choosing a subset of attributes and solely considering those features during the split-making process. This approach can potentially mitigate the correlation among trees and enhance the overall efficacy of the ensemble.

#### Voting

By combining the predictions of base learners, this ensemble learning method generates new features for training sets to improve the desired outcomes [54]. This approach generates the meta-features required for the final prediction by integrating both conventional and sophisticated classifiers. Based on weighted techniques and majority votes, the output of base classifiers is aggregated.

- **Logistic regression**: LR combines multiple logistic regression models to improve overall prediction accuracy [55]. The process involves training logistic regression models iteratively, with each model concentrating on the misclassified instances from the preceding model. This approach exhibits notable efficacy when applied to binary classification tasks.
- **Decision tree**: Boosted DTs sequentially build a series of weak DTs and combine their outputs to create a strong predictive model [56]. It achieves this by repeatedly training DTs in an iterative manner, with each tree concentrating on the most challenging examples from the preceding tree.
- **SVM**: Boosting SVM involves combining the outputs of multiple SVMs to improve classification performance [57]. It trains SVMs iteratively, with each SVM concentrating on the support vectors from the preceding SVM. This method is especially useful for classification tasks involving high-dimensional data.

## Dataset collection and manipulation

We used the Liver Disease Patient Dataset^8^ as the experimental data set, collected from liver patients worldwide and publicly available at the UCI ML repository. This section discusses the details of the dataset and various data preprocessing.

### Dataset description

This data set contains records of a total of 30,691 people, among which 21,917 had liver disease while the rest, 8774 did not have liver ailments. The dataset contains eleven attributes for each record. The first ten attributes are predicate, and the last is a target attribute. Among these, four attributes are of integer type, five are decimal, and two are of categorical type.

Table 2 shows the attribute information such as mean, standard deviation (std), and value range (minimum and maximum). For example, the minimum and maximum values of the total bilirubin (TB) attribute are 0.4 and 75, respectively. And its mean and std values are 3.370 and 6.256, respectively. It has also been observed that less than or equal to 25% of the patients have a TB value of 0.8, while less than or equal to 50% and 75% have a TB value of 1 and 2.7, respectively.

**Table 2:** Summary of attributes of the dataset

Attribute	Description	Measurement	Value range	Mean	Std	25%	50%	75%
Age (AG)	Participant’s age	Years	4–90	44.107	15.981	32	45	55
Gender (GN)	Participant’s gender	Categorical	0 or 1	0.775	0.483	0	1	1
Total bilirubin (TB)	Total bilirubin level in the participant’s blood	mg/dl	0.4–75	3.370	6.256	0.8	1	2.7
Direct bilirubin (DB)	Direct bilirubin level in the participant’s blood	mg/dl	0.1–19.7	1.528	2.870	0.2	0.3	1.3
Alkaline phosphatase (AP)	Alkaline phosphatase level in the participant’s blood	U/L	63-2110	289.075	238.538	175	209	298
Alanine aminotransferase (ALA)	Alanine aminotransferase level in the participant’s blood	U/L	10-2000	81.489	182.159	23	35	62
Aspartate aminotransferase (ASA)	Aspartate aminotransferase level in the participant’s blood	U/L	10-4929	111.470	280.851	26	42	88
Total proteins (TP)	Total protein level in the participant’s blood	g/dl	2.7–9.6	6.480	1.082	5.8	6.6	7.2
Albumin (AL)	Albumin level in the participant’s blood	g/dl	0.9–5.5	3.130	0.792	2.6	3.1	3.8
Albumin and globulin ratio (AGR)	Albumin and globulin ratio in the participant’s blood	g/dl	0.3–2.8	0.943	0.323	0.7	0.9	1.1
Liver disease or not (LD)	If the participant has liver disease or not	Categorical	0 or 1	0.286	0.452	0	0	1

### Exploratory data analysis

**Fig. 3:**
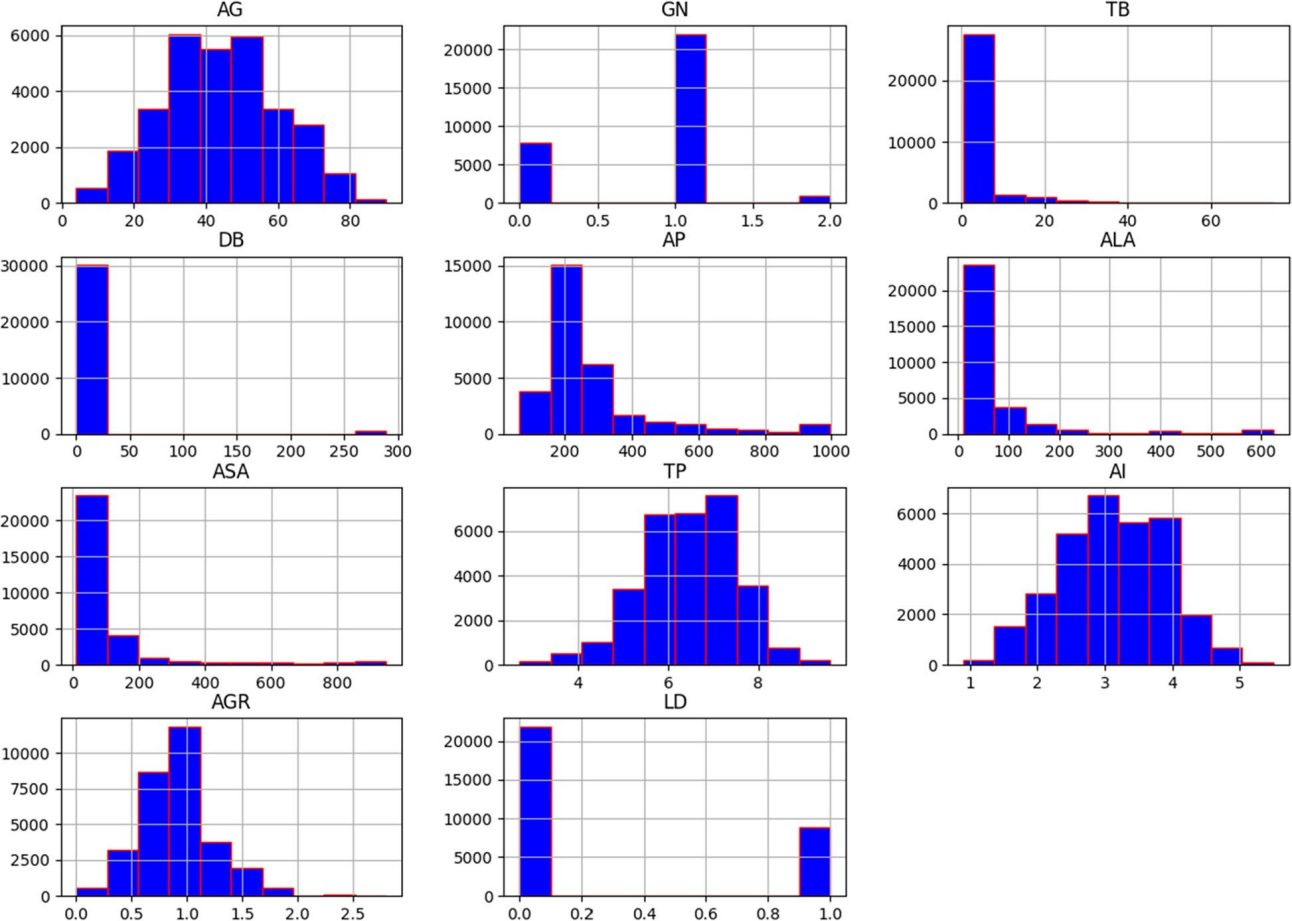
Histogram of dataset attributes

**Fig. 4:**
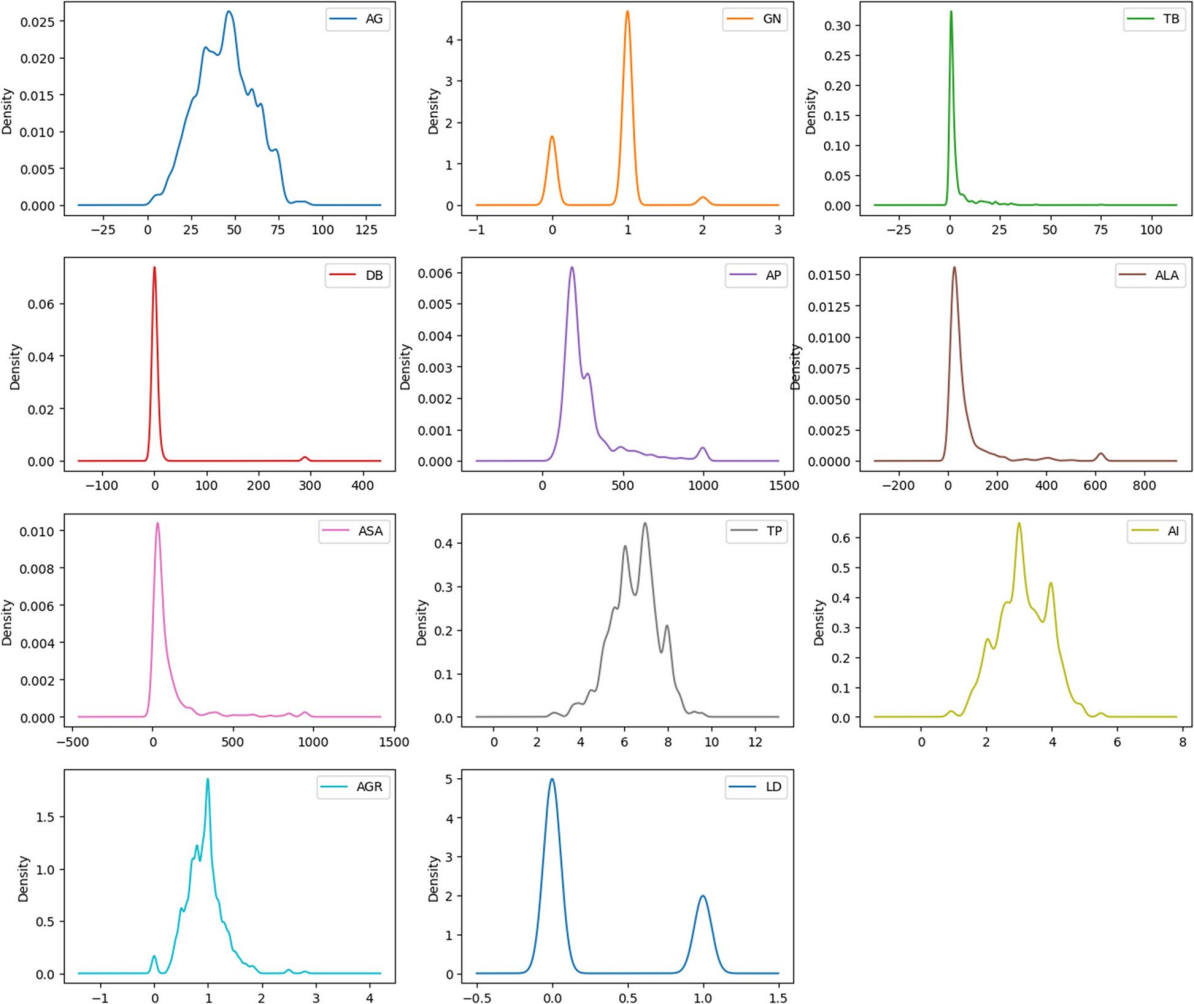
Density plot for KDE (kernel density estimation)

We employed a variety of data visualization techniques to examine and illustrate the data samples’ distribution. The histograms depicted in Fig. 3 are normally distributed and combine the dataset attributes within a given range of values. The X- and Y-axes represent the attribute values and number of patients having those values, respectively. The probability density generated by the KDE method is illustrated in Fig. 4. The X- and Y-axes represent each attribute’s parameter value and probability density function, respectively. It can be observed, for instance, that most patients’ ages in the dataset are between 25 and 65. The IQR approach was exercised to address the presence of outliers in the dataset.

**Fig. 5:**
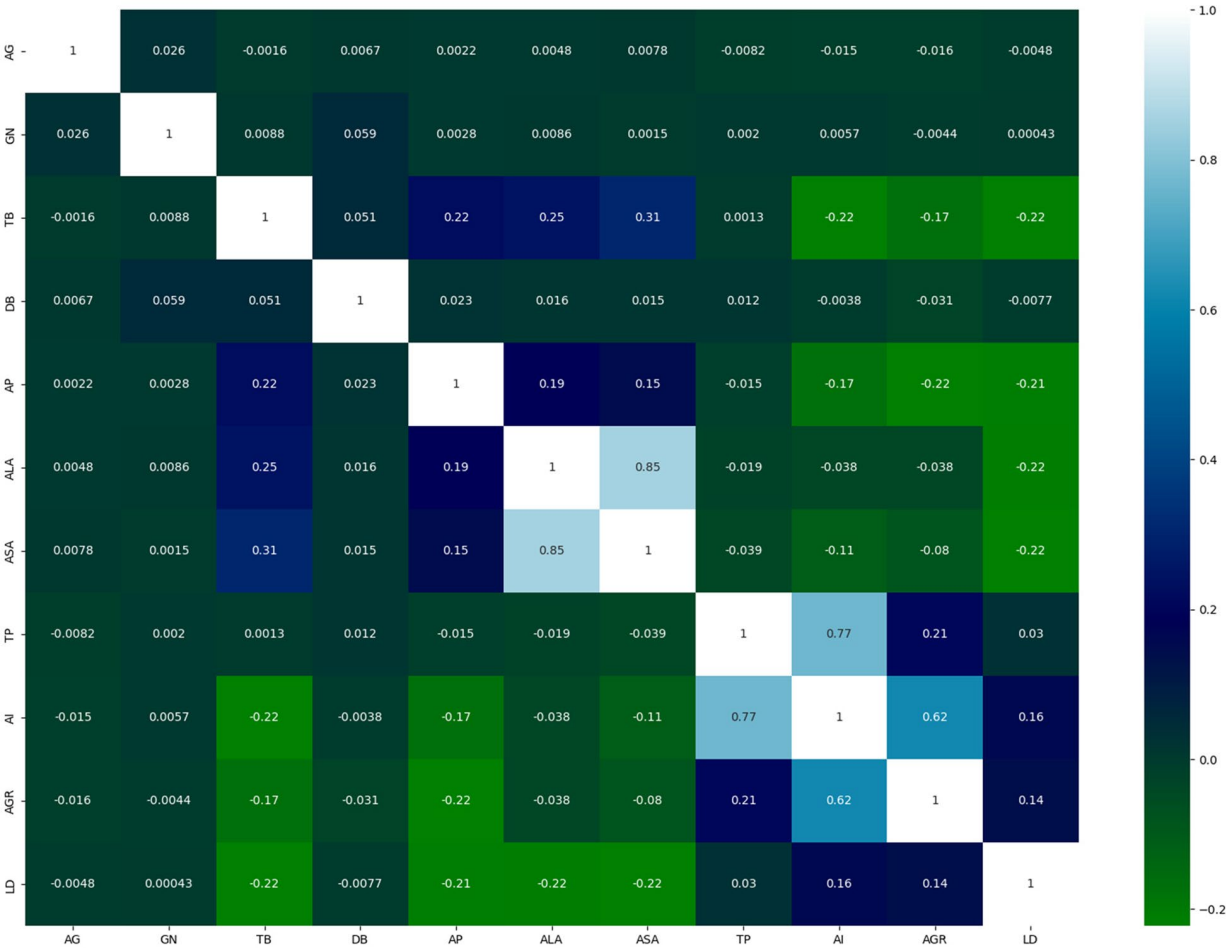
Correlation coefficient analysis

We employed the CCA approach to determine and visualize the relationship between the attributes in the dataset. A substantial correlation or association between the collection of predicate and target attributes indicates a higher-quality dataset. The CCA for the experimental dataset attributes is shown in Fig. 5. The relationship range is bounded by + 1 and − 1 on the X- and Y-axes.

### Data preprocessing

Before applying ML techniques to the model, preparing the data to build a strong and reliable system is important. Several approaches were utilized to handle different data preparation concerns in this study.

#### Outlier detection

**Fig. 6:**
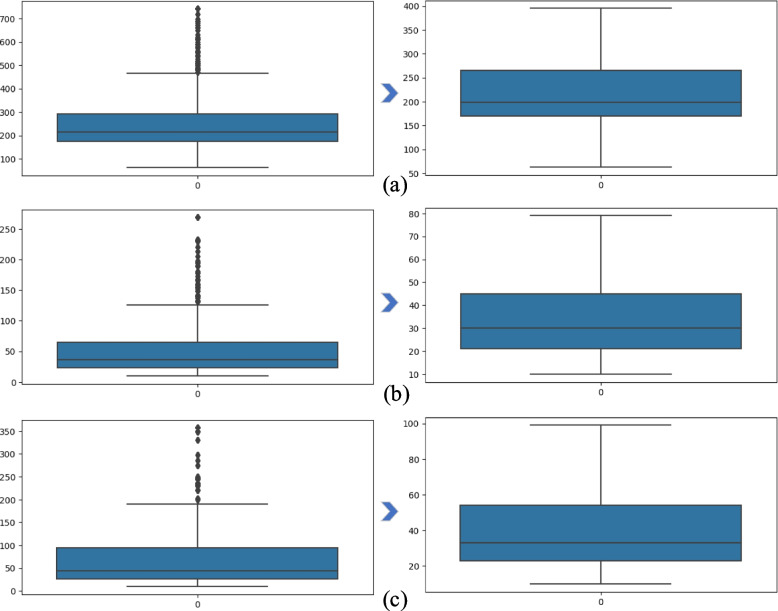
Detecting and replacing outliers in the dataset for (**a**) AP, (**b**) ALA and (**c**) ASA

Identifying outliers and neutralizing them, especially in predictive modelling, is vital in the initial data preparation phase. The process entails identifying data points that exhibit substantial deviation from the other data within the dataset. If outliers are not correctly addressed, they can significantly affect the accuracy of prediction models. We used the IQR method to better visualize outliers in the dataset, if any. We set the threshold of an IQR factor of three for all the features. It was found that the attributes AP, ALA, and ASA had most of the outliers, which is shown in the left column of Fig. 6. The Z-score method, defined by Eq. 1, where x = observed value, µ = mean of the sample, and σ = standard deviation of the sample, was used to replace the outliers. To neutralize the outliers, we set the range for AP, ALA, and ASA as 175–275, 25–45, and 25–55, respectively. The right column of Fig. 6 shows that the outliers of the three attributes are completely removed.

1 $$Z=\frac{x-\mu }{\sigma }$$

#### Missing value imputation

**Fig. 7:**
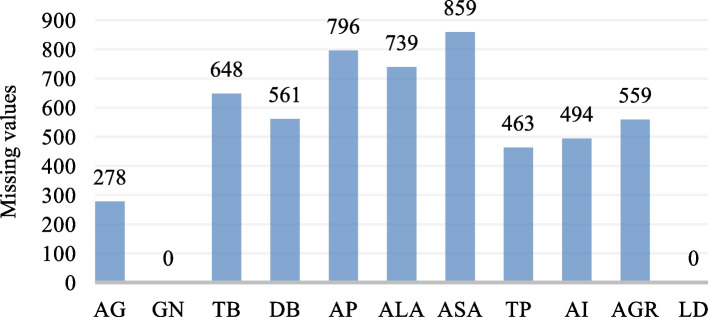
Total number of missing values for each attribute

**Fig. 8:**
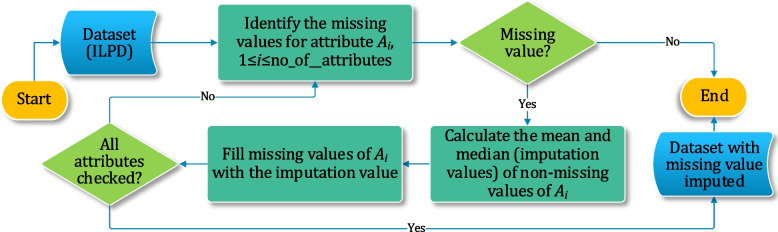
Imputation process of missing values

**Fig. 9:**
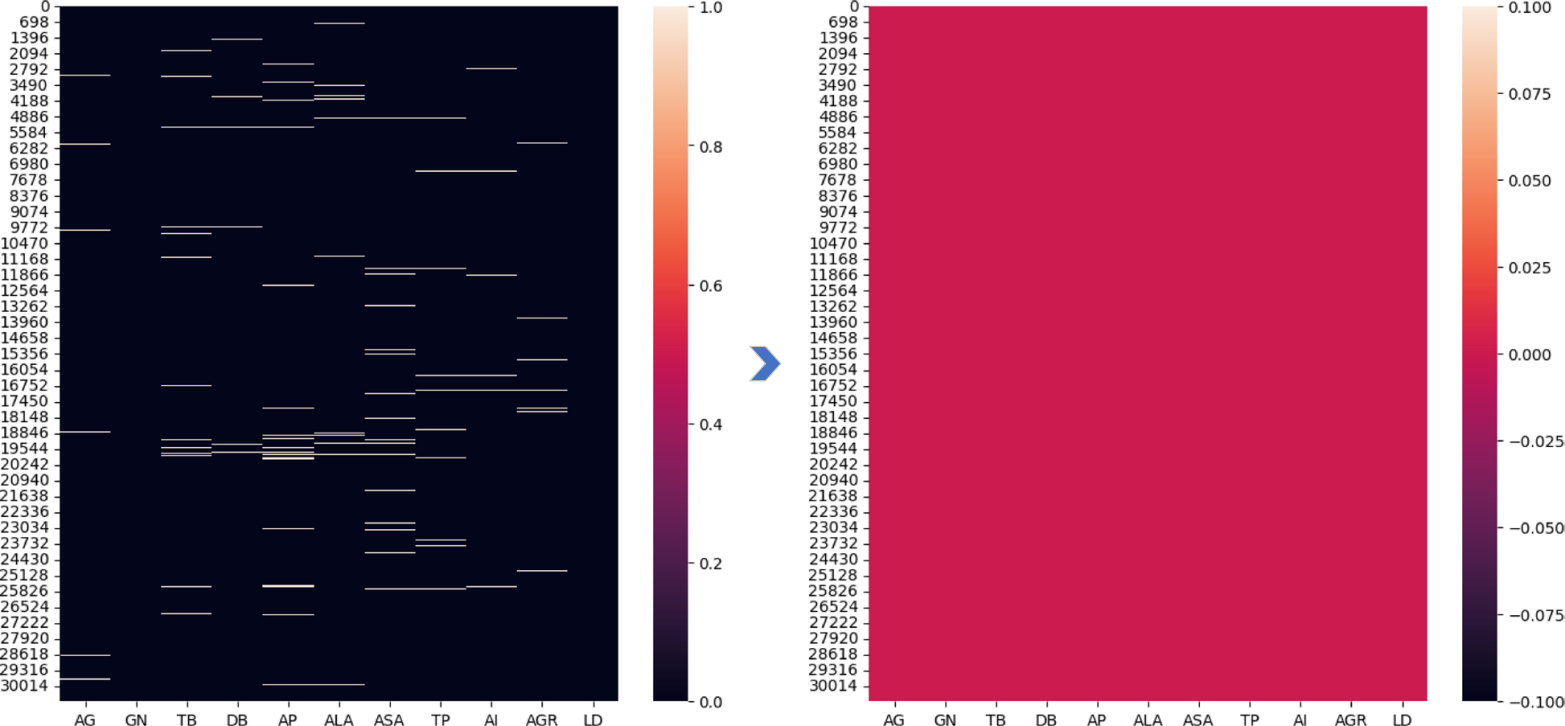
Comparison of missing value identification and replacement. Left panel: before missing value imputation. Right panel: after missing value imputation

Missing value imputation is an important part of predictive modelling because it makes the model work better, reduces bias, improves stability, and improves data representation. This is an important part of preparing the data to ensure that predictive models are accurate, reliable, and useful in many situations. The process entails substituting absent values with credible estimations to guarantee the completeness and coherence of the data before constructing a predictive model. Figure 7 shows the total number of missing values for each attribute in the dataset. We used *isnull()* to find missing values and calculate each attribute’s percentage of null values. Afterwards, we filled in the missing values by the particular attribute’s mean, and median of available values. Figure 8 shows the process of the missing value imputation method. Figure 9 represents the dataset before and after applying the imputation method.

#### Data sampling

**Fig. 10:**
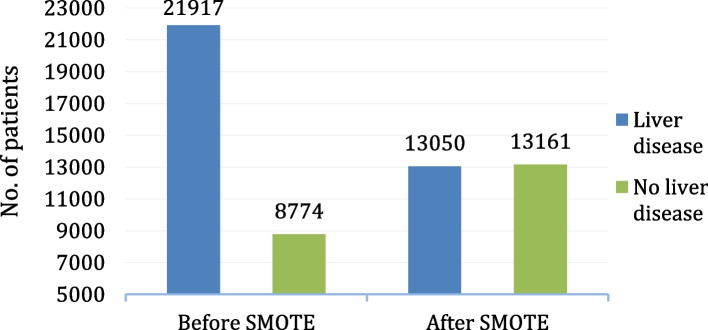
Class balancing of the target variable

If the dataset is imbalanced, ML algorithms perform poorly. The dataset used in this study was significantly skewed toward the positive class (liver disease) rather than the negative class (no liver disease). Originally, out of 30,691 records, 21,917 records were of patients with liver disease, whereas 8779 records were there for patients who did not have liver disease. We balanced the training dataset with respect to the target variable using SMOTE, as shown in Fig. 10.

#### Data normalisation and standardization

For scaling the features, we used the *MinMaxScaler()* function. In our study, we chose this method due to two major advantages. First, it allows to maintain the range of the original features. Second, it is generally robust to outliers because it scales the data based on the minimum and maximum values in the dataset. Outliers are effectively bounded by the range, preventing them from disproportionately affecting the scaling process. Since our dataset originally had outliers, even after removing them, using a min-max scaler would provide a double safeguard.

By applying Eq. 2, we scaled the data values to achieve standardization and batch normalization, with mean and standard deviation values being 0 and 1, respectively.

2 $$N\left(X\right)=\frac{\sum _{i=1}^{N}{x}_{i}-{x}_{min}}{{x}_{max }-{x}_{min} }$$

where, *N*, *X*, *x*_*i*_, *x*_*min*_, and *x*_*max*_ denote the total data sample, i^th^ attribute, the attributes’ mean, the attributes’ sample variance, the sample’s minimum value, and the sample’s maximum value, respectively.

The feature scaling procedure includes normalization, which places the data samples inside a predetermined range that can be determined by the dataset’s type. All of the attributes in our study were scaled from 0 to 1 using min-max as defined by Eq. 3.

3 $${x}_{scaled}=\frac{x-{x}_{min}}{{x}_{max}-{x}_{min}}$$

where *x* is the attribute value, and *x*_*min*_ and *x*_*max*_ denote the minimum and maximum values of *x*, respectively.

## Experiment

This section contains the experimental details of predicting liver disease using ensemble learning algorithms. The details of the experimental setup and configuration are shown in Table 3.

**Table 3:** Hardware and software used to conduct the experiment

Hardware/software	Specification
Processor	Intel(R) Core(TM)- i9-10900 K CPU @3.70 GHz
RAM	64 GB (DDR4)
SSD	500GB (NVMe)
Hard Disk	2 TB (HDD)
Operating System	Windows 11 Pro
Programming Language	Python
Platform	Jupyter Notebook

### Hyperparameter tuning

Hyperparameter tuning is crucial since it governs the behaviour of the training algorithm and has a big impact on the model’s performance assessment. We tuned the hypermeter using the grid search and random search methods to attain optimality in the performance of the suggested model. We preferred these two techniques because they have recently been used in most of the literature and are fairly straightforward to implement. Also, most machine learning frameworks and libraries provide built-in functions or modules for grid and random search. However, we took the search results from the grid search because of better convergence. Grid search also provides better customization and flexibility. Grid search allows for a systematic exploration of different combinations of hyperparameters by defining a grid or a specific set of values for each hyperparameter. This guarantees that all possible options are explored to identify the most optimal values for the hyperparameters. Grid search is deterministic, meaning that it consistently produces the same results when the same hyperparameters and data are utilized. This attribute enables transparent testing and assessment by ensuring that outcomes are easy to reproduce and compare. Table 4 displays the specifics of the hyperparameters for every method. In our experiment, we discovered that the optimal values for each parameter in the corresponding method were those that were listed.

**Table 4:** Hyperparameters for the boosting algorithms

Algorithm		Hyperparameters
Boosting	XGB	XGBClassifier (learning_rate = 0.1, n_estimators = 1000, max_depth = 5, min_child_weight = 6, ‘reg_alpha’: 60.0, subsample = 0.6, colsample_bytree = 0.8, ‘gamma’: 4.20)
	GB	GradientBoostingClassifier(random_state = 45, learning_rate = [0.1, 2, 5], n_estimators = 5000, max_depth = 4, weight = 6, verbose = 1)
	LGBM	LightGBMClassifier (boosting_type = ‘lgbm’, random_state = 45, learning_rate = 0.1, n_estimators = 1000, max_depth = 2, min_child_samples = 250, silent = True, n_jobs = 6)
Bagging	BDT	BaggingDecisonClassifier(base_estimator = None, bootstrap = False, bootstrap_features = True, n_estimators = 500, n_jobs = -1, oob_score = False, random_state = 42, verbose = 0)
	RF	RandomForestClassifier (n_estimators = 1000, criterion = ‘gini’, max_depth = None, min_samples_split = 2, min_samples_leaf = 1, max_features = 16, bootstrap = True, random_state = 42)
	ET	ExtraTreesClassifier (n_estimators = 1000, criterion = ‘gini’, max_depth = 1000, min_samples_split = 10, min_samples_leaf = 2, max_features = 10, bootstrap = 2, random_state = 42)
Voting	LR + DT + SVM	StackingClassifier(estimators = [(‘lr’, LogisticRegression(),dt, DecisionTree(), ‘svm’, SVC(probability = True)], voting = ‘soft’), params = {‘lr__C’: [1.0, 100.0], ‘svm__C’: [2, 3, 4], estimator = eclf, param_grid = params, cv = 2)

### Cross validation

K-fold cross-validation is commonly employed to mitigate bias in the dataset. This approach involves dividing the dataset into *k* subsets of roughly equal size, referred to as “folds”. The experiment involved implementing *k*-fold cross-validation on the training dataset. We tested with different values of *k* from 4 to 12. For *k* = 4 to 9, we found overfitting for most of the considered models, while values 11 and 12 of *k* introduced underfitting to the models. Our training and testing evaluation for all the models indicated the best balance between overfitting (smaller *k* values) and underfitting (higher *k* values) is *k* = 10.

### Feature importance and selection

**Fig. 11:**
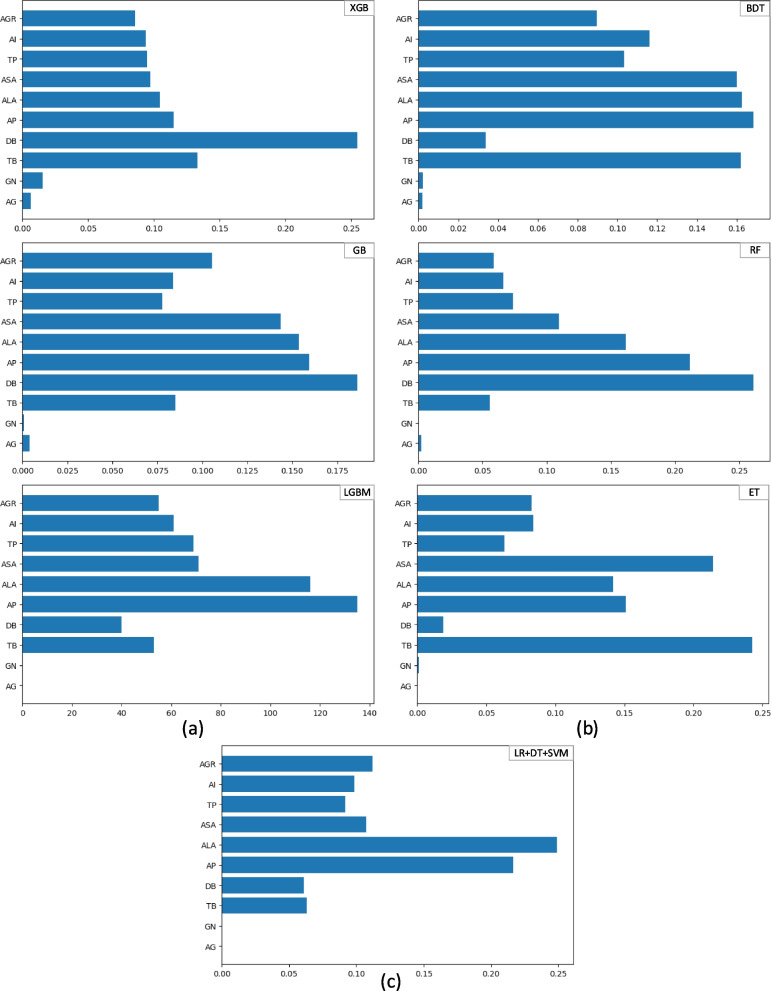
Feature importance for prediction using (**a**) boosting, (**b**) bagging, and (**c**) voting

The feature significance procedure ranks the predictor variables (input attributes) according to how well they help predict the target variable (output feature). This stage is critical for generating more accurate predictions for ML and ensemble learning models. We used the feature significance score (F-score), a metric that determines the frequency with which an attribute is utilized for splitting during the training process. Figure 11 illustrates the contributions made by each predicate parameter utilized in this investigation. The features and their degree of significance are plotted on the Y- and X-axis, respectively. As seen in the figure, DB, AP, ALA, and ASA are the most significant factors that lead to an accurate prognosis of liver disease; on the other hand, the demographic parameters (GN and AGE) are the least significant factors that influence the prediction are liver disease. We also checked for potential collinearity among features using the VIF method and found that none of the attributes had high collinearity. The observed VIF value lay between 0 and 4, eliminating the possibility of overfitting.

## Results and performance evaluation

This section discusses the performance of the designed prediction model for considered ensemble algorithms using various performance indicators.

### Evaluation metrics

Evaluation metrics are used to assess the performance of a model on a problem statement. Different evaluation metrics are used depending on the problem type and the data’s nature [58]. In this study, the experimental findings for the presented model are evaluated using various performance metrics, as summarised in Table 5 [59], where, true positive (TP): the patient has liver disease, and the model predicts liver disease, true negative (TN): the patient does not have liver disease and the model predicts negative, false positive (FP): the patient does not have liver disease but the model predicts liver disease, and false negative (FN): the patient has the liver disease but the model predicts negative.

The evaluation of the ensemble algorithms’ predictive capability is generally conducted across multiple levels by employing the ROC curve. By analyzing the ROC curve, we can determine how well the models can distinguish between the TPR and FPR. The model’s ability to differentiate between the two classes is indicated by a higher ROC curve [60]. The AUC is also used to measure how well two classes can be separated. Generally, a good separability measure has an AUC close to 1, whereas a poor separative measure has an AUC close to 0. A value of 0.5 suggests the model is not classifying well.

**Table 5:** Performance evaluation metrics

Metrics	Calculation	Description
Accuracy	$\frac{TP + TN}{TP + TN + FP + FN}$	The number of instances when both LD and NLD are correctly predicted out of the total prediction by the model. A higher accuracy suggests that the model is better at correctly classifying both individuals with LD and those without LD.
Precision	$\frac{TP}{TP + FP}$	The number of instances when the patient actually has LD out of the total true LD and false LD prediction made by the model. When the precision is higher, the model is more reliable in identifying individuals with LD, and there are fewer cases where individuals are incorrectly classified as positive when they are actually negative.
Recall/TPR	$\frac{TP}{TP + FN}$	The number of instances when the patient actually has LD out of the total instances predicted by the model for true LD and false NLD. A higher recall suggests that the model is better at capturing cases of LD, meaning it is less likely to miss individuals who are actually suffering from the condition.
F1-score	$\frac{2\times TP}{2\times TP + FP+ FN}$	The harmonic mean of the recall and precision. A higher F1-score suggests the model has a better balance between precision and recall, meaning it is better at correctly identifying both the positive and negative instances of LD.
Specificity	$\frac{TN}{TN + FP}$	The number of instances when the patient actually does not have LD out of the total instances predicted by the model for true NLD and false LD. A higher specificity suggests that the model is better at avoiding false alarms of LD.
Macro average (MA)	$\frac{1}{4}\sum\limits _{c=0}^{3}{A}_{c}^{m}$	The arithmetic mean of the individual class for precision, recall, and f1-score, where *c* denotes classes 0 to 3 and *m* denotes either precision or recall or F1-score.
Weighted average (WA)	$\sum\limits _{c=0}^{3}{w}_{c}^{m}\times \frac{1}{4}\sum\limits _{c=0}^{3}{A}_{c}^{m}$	The arithmetic mean of the individual class multiplied by respective weights for precision, recall, and F1-score, where ${w}_{0}+{w}_{1}+{w}_{2}+{w}_{3}=1$.
Negative predicted values (NPV)	$\frac{TN}{TN + FN}$	The number of instances when the patient actually does not have LD out of the total true NLD and false NLD prediction made by the model. A higher NPV implies that the model is better at ruling out LD in individuals who are actually disease-free. This indicates a higher confidence level in the model’s ability to accurately identify individuals who do not have LD, reducing the likelihood of missed diagnoses and ensuring that fewer individuals are mistakenly classified as healthy when needing medical attention.
Matthews corelation coefficient (MCC)	$$\frac{TP \times TN-FP\times FN}{\sqrt{\begin{array}{c}\left(TP+FP\right)\times \left(TP+FN\right)\times \\ (TN+FP)\times (TN+FN)\end{array}}}$$	Indicates a balanced performance of the model in predicting both LD and NLD. A higher MCC suggests that the model’s predictions are more consistent with the true labels, and there is a stronger agreement between the model’s predictions and the actual outcomes.
False-positive rate (FPR)	$\frac{FP}{FP + TN}$	The number of instances when the model falsely predicts LD out of the total instances predicted by the model for false LD and true NLD. A lower FPR in LD prediction indicates that the model has a better ability to correctly identify individuals without LD, reducing the likelihood of false alarms and improving the overall accuracy of the diagnostic process. Reducing the FPR is crucial in medical diagnosis because it helps minimize unnecessary stress, follow-up tests, and treatments for individuals who are actually disease-free.
False-negative rate (FNR)	$\frac{FN}{TP + FN}$	The number of instances when the model falsely predicts NLD out of the total instances predicted by the model for true LD and false NLD. A lower FNR in LD prediction indicates that the model has a better ability to correctly identify individuals with liver disease, reducing the likelihood of missed diagnoses. Reducing the FNR is crucial in medical diagnosis because it helps ensure that individuals who have LD are correctly identified and receive timely treatment.
False discovery rate (FDR)	$\frac{FP}{FP + TP}$	The number of instances when the model falsely predicts LD out of the total instances predicted by the model for false LD and true LD. When the FDR is lower, fewer individuals are incorrectly classified as having LD when they are actually healthy. Lowering the FDR is crucial in medical diagnosis because it helps reduce unnecessary stress, follow-up tests, and treatments for individuals who are actually disease-free. By minimizing FP predictions, the model becomes more reliable in identifying true cases of LD.
Misclassification rate (MCR)	$\frac{FP + FN}{TP + TN + FP + FN}$	The number of instances when both LD and NLD are incorrectly predicted out of the total prediction by the model. A lower MCR indicates that the model is performing well in accurately identifying cases of LD while minimizing incorrect classifications. It reflects a higher level of effectiveness and reliability in the diagnostic process.
Runtime	-	Amount of time (in minutes) required to execute the algorithm.

### Comparing bagging, boosting and voting methods

**Fig. 12:**
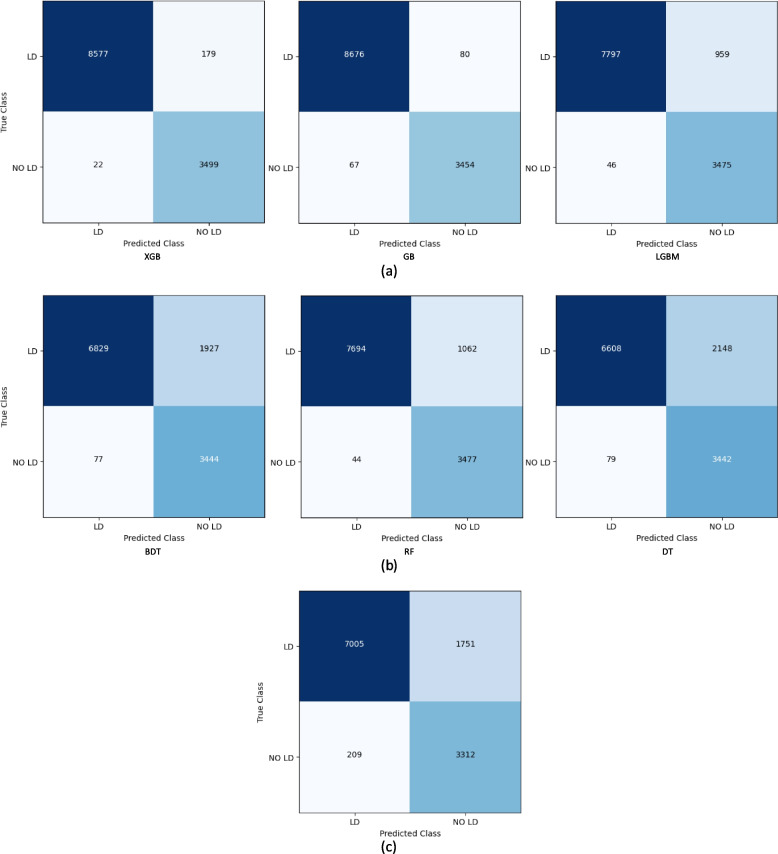
Confusion matrices of (**a**) boosting, (**b**) bagging and (**c**) voting algorithms

**Fig. 13:**
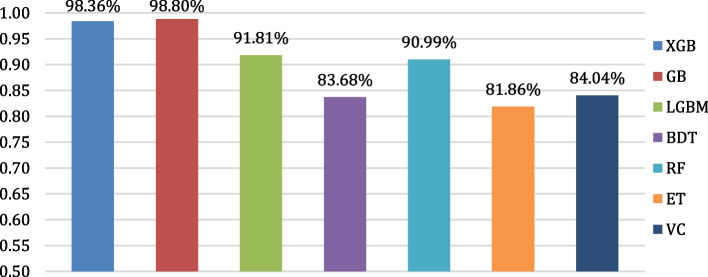
Accuracy comparison of the considered algorithms

**Fig. 14:**
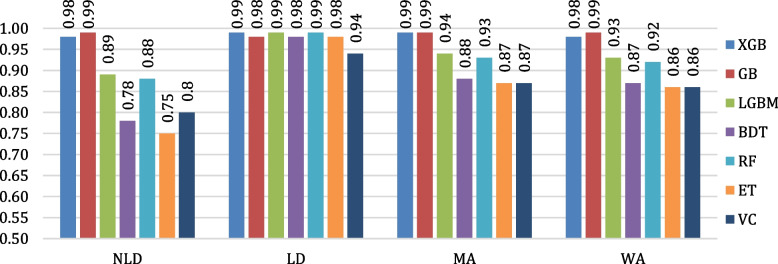
Comparison of precision values of the considered algorithms

**Fig. 15:**
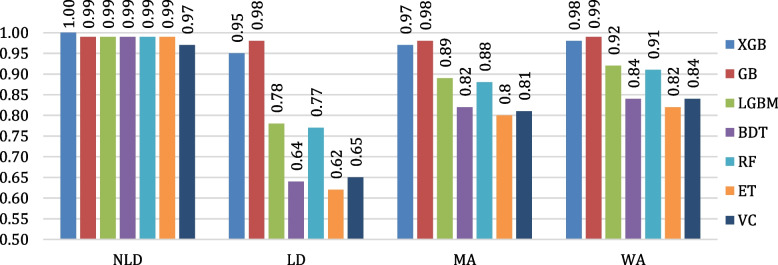
Comparison of recall values of the considered algorithms

**Fig. 16:**
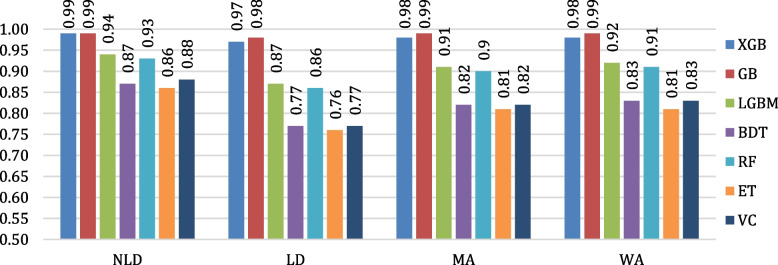
Comparison of F1-score values of the considered algorithms

**Fig. 17:**
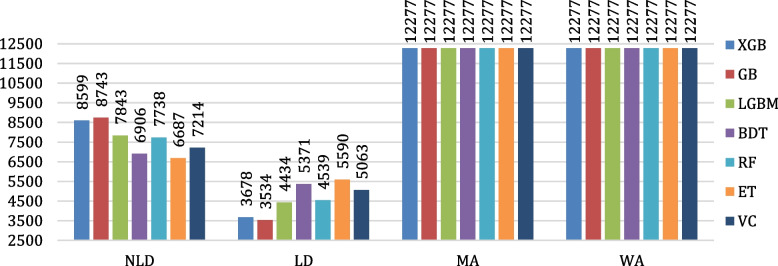
Comparison of support values of the considered algorithms

The evaluation of the algorithms’ classification performances is conducted by means of confusion matrices. The confusion matrices of all the considered algorithms are shown in Fig. 12. Figure 13 depicts the testing accuracies of all algorithms. As per our experiment, GB outperformed other algorithms by attaining the maximum accuracy rate of 98.80%, followed by XGB and LGBM, while ET attained the lowest accuracy of 81.86%. The precision, recall, F1- score, and support of the algorithms are shown in Figs. 14, 15, 16 and 17. In most cases, GB performed best. The nearest competitor was found to be XGB, whereas LGBM and RF had fair overall performance.

**Fig. 18:**
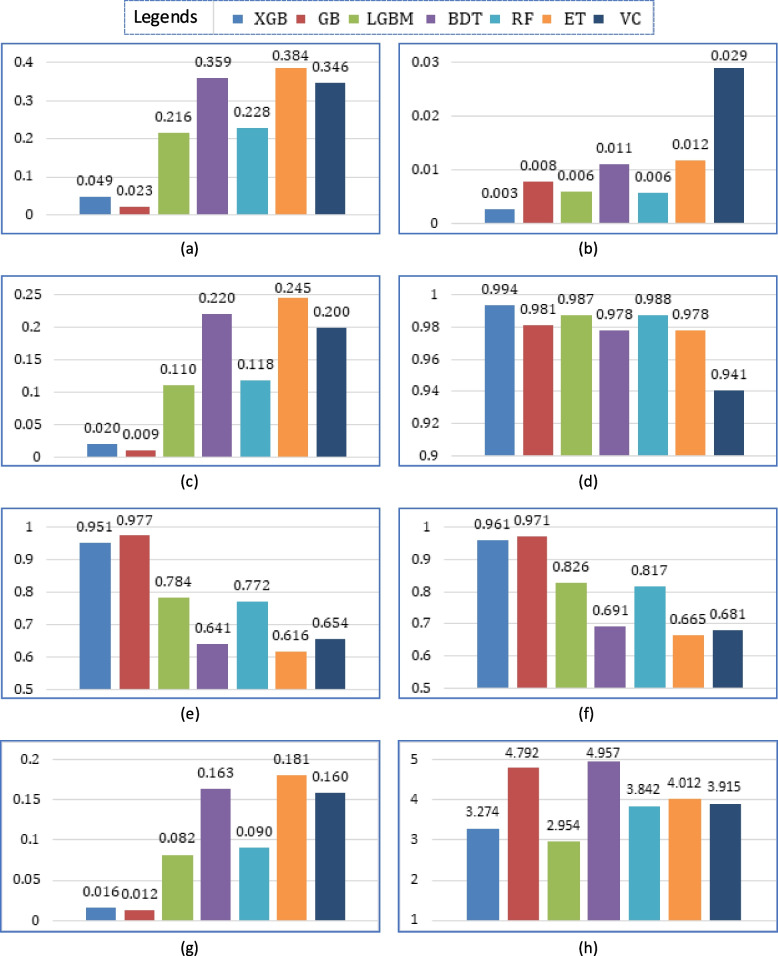
Comparisons of (**a**) FPR, (**b**) FNR, (**c**) FDR, (**d**) NPV, (**e**) specificity, (**f**) MCC, (**g**) MCR, and (**h**) run time of the considered algorithms

The other comparing measurements (FPR, FNR, FDR, NPV, specificity, MCC, MCR, and run time) are shown in Fig. 18. It can be observed that GB excels in FPR, FDR specificity, MCC, and MCR, whereas XGB betters in FNR and NPV. In only one parameter (RT), GB fails. It took the second most time (after BDT), while LGBM took the least time.

**Fig. 19:**
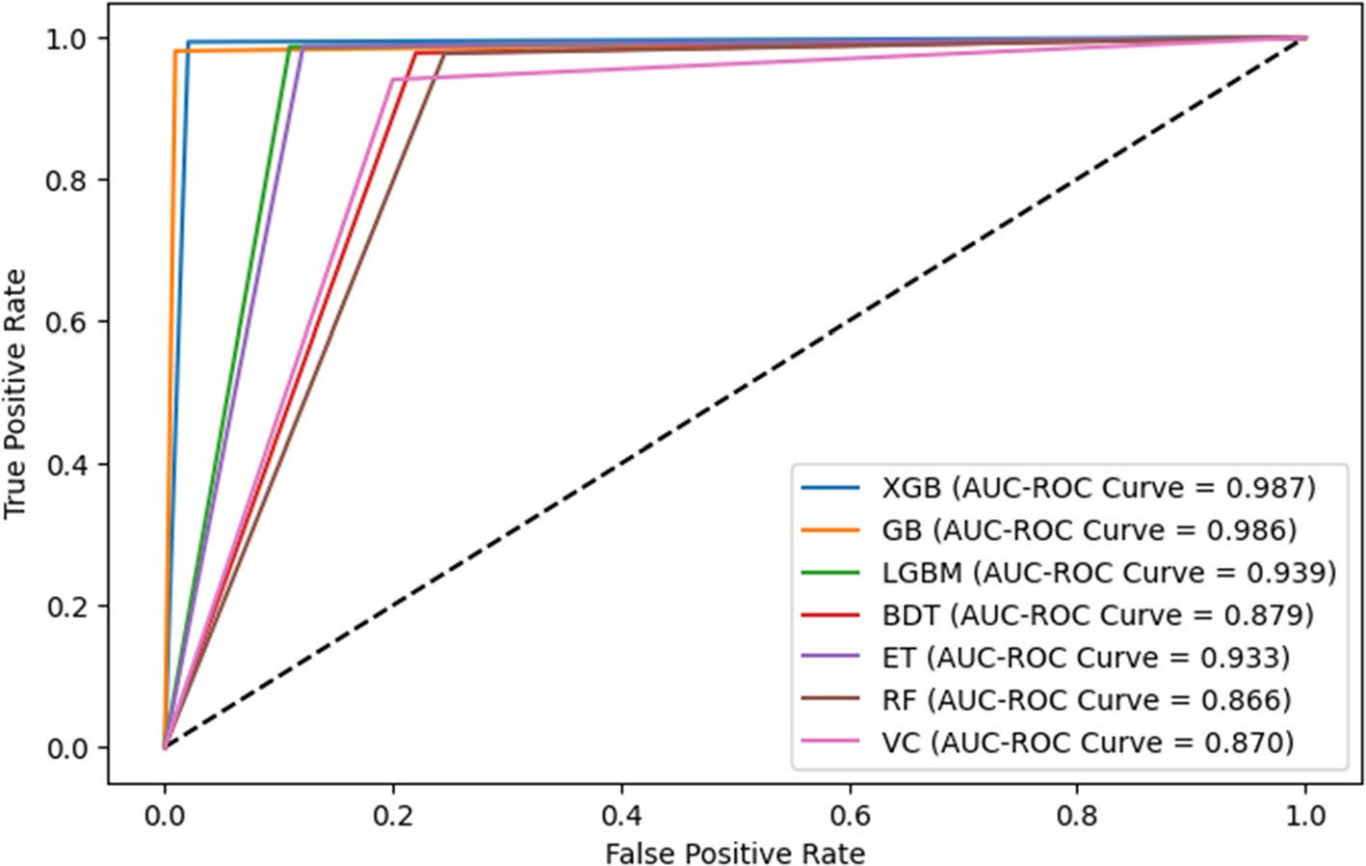
The AUC-ROC curves for the considered algorithms

The AUC-ROC curves for the considered algorithms are shown in Fig. 19. According to the curves, GB (0.986) performed marginally inferior to the top performer, XGB (0.987) the best, while RF (0.866) performed the worst of the algorithms tested.

### Comparative analysis with literature

To establish the performance of our model, we compared it with several similar research papers in respect of various metrics, as shown in Table 6. Given that GB demonstrated superior overall performance in predicting liver disease in our experiment, we compared the outcomes achieved exclusively with GB. The better performance attained by our model can be ascribed to the implemented methodologies, which include data imputation to account for missing values, identification and substitution of outliers, and efficient data normalization and standardization.

**Table 6:** Comparing the proposed model with recent literature

Research work	Algorithms considered	Dataset used	Highest accuracy (%)	Precision (%)	Recall (%)	F1-score (%)	Specificity (%)	AUC/-ROC (%)	Negative predicted values (%)	MCC (%)	False-positive rate	False-negative rate	False discovery rate	Misclassification rate	Run time (mins.)
Amin et al. [28]	LR, KNN, RF, SVM, MLP, voting	ILPD	88.10 with RF	85.33	92.3	88.68	-	88.20	93.00	-	-	0.0700	0.1467	0.1190	3.337
Afrin et al. [29]	LR, DT, RF, AdaBoost, KNN, LDA, GB, SVM	ILPD	94.29 with DT	92	99	96	-	-	99.00	-	-	0.0100	0.0800	0.0571	-
Bulucu et al. [36]	RF, J48, GB, AdaBoost, LGBM	UCI dataset	98.8 with LGBM	98.1	99.4	-	-	-		-	-	0.0060	0.1960	0.0120	-
Dritsas and Trigka [30]	NB, SVM, LR, ANN, kNN, J48, RF, RT, RepTree, RotF, AdaBoostM1, stacking, bagging, voting	ILPD	80.1	80.4	80.1	80.1	-	88.4	80.10	-	-	0.1990	0.1960	0.1990	-
Gupta et al. [40]	LR, DT, RF, KNN, XGB, LGB	ILPD	63 with LGB and RF both	64 with RF	63 with RF	63 with LGB and RF both	-	-	63.00	-	-	0.3700	0.3600	0.3700	-
Hameed et al. [41]	RF, SVM, LR, DT, AdaBoost, GB, KNN	ILPD	80.30 with RF	80.30	80.26	80.20	-	-	80.30	-	-	0.1974	0.1970	0.1970	-
Kuzhippallil et al. [32]	LR, kNN, DT, RF, GB, AdaBoost, XGB, LGBM, stacking	ILPD	86 XGB, LGBM	86 XGB	86 XGB	86 XGB	-	-	86.00	-	-	0.1400	0.1400	0.1400	0.191
Nahar et al. [31]	AdaBoost, LogitBoost, RF, bagging (RepTree and J48)	ILPD	71.53 with LogitBoost	83.60	56.45	-	-	72.20	56.45	-	-	0.4355	0.1640	0.2847	-
Naseem et al. [33]	A1DE, MLP, NB, kNN, SVM, J48, CHIRP, CDT, forest-PA, RF	BUPA	72.17% with RF	62.07	68.7	65.22	74.30	-	79.50	42.28	0.2570	0.3130	0.3793	0.2783	-
		SanikaVT	71.36% with SVM	100	71.36	83.28	-	-	0	-	-	0.2864	0	28.64	-
Quadir et al. [34]	GB, XGB, bagging, RF, ET, stacking	ILPD	93.15 with stacking	80.76	94.59	87.13	74.22	84.41	94.59	-	0.2578	0.0541	0.1924	0.0685	-
Dalal [35]	Hybrid XGB	ILPD	93.65	-	-	-	-	98.70	-	-	-	-	-	0.0635	-
Zhao et al. [42]	SVM, GP, RF, XGB, bagging	BUPA	80.35 with RF	68.75	38.82	49.62	-	-	38.82	-	-	0.6118	0.3125	0.1965	-
Our paper	XGB, LGBM, GB, BDT, RF, ET, LR, DT, SVM	LDPD	98.80 with GB	98.50	98.50	98.50	97.74	98.60	98.10	97.08	0.0226	0.0077	0.0091	0.0120	4.792

## Conclusions

Liver disease causes two million deaths annually and affects many more patients worldwide. In this paper, we designed ensemble learning based models and evaluated them to find the best model that would accurately predict liver disease. We examined the effectiveness of three ensemble learning approaches: boosting, bagging and voting. Furthermore, for each approach, we considered three algorithms, i.e., gradient boosting, XGB, and LGBM for boosting, RF, ET and BDT for bagging and LR, DT and SVM for voting.

GB demonstrated the highest level of performance in the experiment, attaining an accuracy rate of 98.80%. However, in some parameters (e.g., precision (liver disease), recall (no liver disease), false negative rate, negative predicted values, and ROC), XGB performed better. The performances of LGBM and BDT were also fair. LGBM was the fastest to execute, while GB was the slowest. Our proposed model was compared with several similar works, in which it was found to outperform them.

Due to their simplicity and convenience, we used mean and median methods to fill in the missing values. However, the straightforwardness of these methods brings some obvious limitations, such as loss of variability, distortion of relationships, introduction of biases, underestimation of uncertainty, and sensitivity to missingness patterns. To mitigate these limitations, alternative imputation methods that consider the underlying characteristics of the data and the missingness mechanism can be explored. Also, we used the SMOTE method to balance the dataset, which may introduce issues like overfitting, data leakage, noise amplification, parameter-sensitivity, and imbalanced feature representation. Though we carefully evaluated the impact of SMOTE on the ensemble models’ performance and took measures such as feature selection, alternative techniques, such as modified versions of SMOTE (e.g., borderline-SMOTE, ADASYN) or other data resampling methods, can be explored to address class imbalance while minimizing the potential drawbacks associated with the SMOTE method.

To broaden the applicability of this study, the proposed method may be extended to encompass additional healthcare datasets that possess similar characteristics. In subsequent research, investigating deep learning techniques might result in improved liver disease detection and prediction. The developments in deep learning and advanced machine learning may lead to more precise and effective medical treatments.

## Data Availability

No datasets were generated or analysed during the current study.

## Footnotes

1 https://www.bupa.com/.

2 https://archive.ics.uci.edu/ml/datasets/liver+disorders.

3 https://archive.ics.uci.edu/ml/datasets/ILPD+(Indian+Liver+Patient+Dataset).

4 https://www.kaggle.com/c/liver-patient-dataset.

5 https://archive.ics.uci.edu/ml/datasets/liver+disorders.

6 https://github.com/SanikaVT/Liver-disease-prediction.

7 https://archive.ics.uci.edu/ml/datasets/HCV+data.

8 https://www.kaggle.com/datasets/abhi8923shriv/liver-disease-patient-dataset/data.
